# Lack of Association between Cytotoxic T-lymphocyte Antigen 4 (*CTLA-4*) -1722T/C (rs733618) Polymorphism and Cancer Risk: From a Case-Control Study to a Meta-Analysis

**DOI:** 10.1371/journal.pone.0094039

**Published:** 2014-04-07

**Authors:** Weifeng Tang, Hao Qiu, Heping Jiang, Bin Sun, Lixin Wang, Jun Yin, Haiyong Gu

**Affiliations:** 1 Department of Cardiothoracic Surgery, Affiliated People's Hospital of Jiangsu University, Zhenjiang, China; 2 Department of Microbiology and Immunology, Medical School of Southeast University, Nanjing, China; 3 Department of Respiratory Disease, Affiliated Jintan People's Hospital of Jiangsu University, Jintan, China; The University of Hong Kong, Hong Kong

## Abstract

**Background:**

The association between cytotoxic T-lymphocyte antigen 4 (*CTLA-4*) gene -1722T/C polymorphism (rs733618) and cancer has been widely assessed, and a definitive conclusion remains elusive. We first performed a hospital based case-control study to measure this association of esophageal cancer with *CTLA-4* -1722T/C polymorphism in Han Chinese population, and then carried out a meta-analysis to obtain a comprehensive evaluation for this issue.

**Methodology/Principal Findings:**

This case-control study involved 629 esophageal squamous cell carcinoma (ESCC) cases and 686 age and gender well matched cancer-free controls. PCR-LDR (polymerase chain reaction-ligase detection reactions) method was used to identify genotypes. Meta-analysis was conducted by STATA (v12.0) software. This case-control study showed no significant difference in the genotype and allele distributions of *CTLA-4* -1722T/C polymorphism between esophageal cancer cases and control subjects, in accord with the findings of the further meta-analysis in all genetic models. Evidence of large heterogeneity was observed among all eligible studies in the recessive model. Further subgroup analyses by ethnicity, cancer type and system, detected null associations in this meta-analysis.

**Conclusion:**

This case-control study and the further meta-analysis, failed to identify the association between *CTLA-4* -1722T/C polymorphism and cancer risk.

## Introduction

It is estimated that about 12.7 million multiple cancer cases and 7.6 million cancer deaths have occurred in 2008 worldwide, with more than half of the cases and about two-thirds of the deaths in the developing countries [1]. The evidence is mounting that cancer is a complex disease results from interactions between multiple genetic backgrounds and environmental factors [2], [3]. Of late, a number of studies demonstrate that genetic variants of the genes that regulate the activation and proliferation of T lymphocytes and nature killer (NK) cells may influence cancer risk [4], [5]. In the last decade, single nucleotide polymorphisms (SNPs) have been extensively investigated, and many studies have examined the hypothesis that genetic variants of the immune genes may be relevant to the risk of a variety of cancers [6], [7].

Cytotoxic T-lymphocyte antigen 4 (CTLA-4), also named CD152, is a member of the immunoglobulin superfamily. CTLA-4 is expressed mainly on activated T cells, acts as a vital restraining regulator of T-cell proliferation and activation, and induces Fas-independent apoptosis of activated T cells to further inhibit immune function of T-cell [6], [8]. Blocking CTLA-4 function and enhancing T cell activation, several different types of malignant neoplasms in tumor-transplanted mice were inhibited or cured, and owned long-lasting antitumor immunity [9]. It suggests that CTLA-4 plays an important role in carcinogenesis. *CTLA-4* gene is located on chromosome 2q33, and is composed of four exons that encode several functional domains of the CTLA-4 protein and possess several vital SNPs, such as the +49A/G (rs231775), -318C/T (rs5742909), CT60G/A (rs3087243), -1661A/G (rs4553808), and -1722T/C (rs733618) SNPs, etc [6], [10].

A meta-analysis showed that *CTLA-4* +49A/G polymorphism may be a risk factor for cancer, whereas -318C/T and +6230G/A (CT60) polymorphisms were lack of association with cancer [4]. Of late, Geng and colleagues reported a meta-analysis with a negative result on the association between *CTLA-4* -1722T/C polymorphism and cancer risk [11]. Linkage disequilibrium (LD) plot of *CTLA-4* (involving rs733618, rs4553808, rs5742909, rs231775 and rs3087243) was generated using Haploview 4.2 program and the results suggest that −1661A/G (rs4553808) and −318C/T (rs5742909) are in high LD; the others are in low LD [11]. The *CTLA-4* -1722T/C polymorphism has not been investigated in esophageal cancer. To further investigate this potential relationship, we decided to evaluate the association of *CTLA-4* -1722T/C polymorphism with esophageal cancer risk in a hospital based case-control study, and then performed a comprehensive meta-analysis to derive a more precise result.

## Materials and Methods

### Subjects

This hospital-based case–control study included 629 sporadic esophageal squamous cell carcinoma (ESCC) cases and 686 cancer-free subjects consecutively recruiting from the Affiliated People's Hospital of Jiangsu University and Affiliated Hospital of Jiangsu University (Zhenjiang City, Jiangsu Province, China), between October 2008 and December 2010. All recruited subjects were local residents of Han Chinese population, and all ESCC subjects were diagnosed by surgical resection and pathologic examination. The ESCC subjects who had a history of personal malignant tumor or autoimmune disorder, or had undergone radiotherapy or chemotherapy were excluded. Ethnicity, gender and average age (±5 years) of the controls were well matched to esophageal cancer cases. The control individuals were selected from the two hospitals for cure of fracture. At recruitment, this hospital based case-control study was approved by the Ethics Committee of Jiangsu University (Zhenjiang, China). Information of all subjects was collected from a structured questionnaire which was administered by two experienced research doctors. The information of demographic data (e.g. age, gender) and related risk factors (such as, tobacco use and alcohol consumption) is listed in **Table 1**. Each subject signed the written informed consent and donated 2-ml sample of peripheral blood.

**Table 1:** Distribution of selected demographic variables and risk factors in ESCC cases and controls.

Variable	Cases (n = 629)		Controls (n = 686)			*P* a
	n	%		n	%	
**Age (years)** mean ± SD	62.85 (±8.13)			62.58 (±7.89)		0.541
**Age (years)**						0.155
<63	310	49.28		365	53.21	
≥63	319	50.72		321	46.79	
**Sex**						0.185
Male	444	70.59		461	67.20	
Female	185	29.41		225	32.80	
**Tobacco use**						**<0.001**
Never	355	56.44		499	72.74	
Ever	274	43.56		187	27.26	
**Alcohol use**						**<0.001**
Never	428	68.04		526	76.68	
Ever	201	31.96		160	23.32	

a Two-sided *χ* ^2^ test and student *t* test; Bold values are statistically significant (*P*<0.05).

### DNA extraction, SNP selection, and genotyping

Blood samples were collected with ethylenediamine tetra-acetic acid (EDTA) anticoagulant vacutainer tubes (BD Franklin Lakes NJ, USA). Genomic DNA was extracted from lymphocytes using the QIAamp DNA Blood Mini Kit (Qiagen, Berlin, Germany) and DNA samples were frozen at −80°C. Genotyping of *CTLA-4* -1722T/C polymorphism was carried out using the polymerase chain reaction-ligase detection reactions (PCR-LDR) method [12]. The Shanghai Biowing Applied Biotechnology Company provides technical support for genotyping. One hundred and sixty samples were randomly selected and reciprocally tested with directly sequencing for quality control, and the reproducibility were 100%. The primers of directly sequencing used for *CTLA-4* -1722T/C genotyping were as follows: F: 5' GCAATAACAACCTAATGGGCAC 3'; **R**: 5' ACTTCCACAGGCTGAACCACT 3' (**Figure S1**).

### Statistical analysis

Chi-square test (*χ* ^2^) was conducted to measure the differences in the distributions of genotypes, demographic characteristics and selected variables between esophageal cancer cases and controls. Genotype frequencies of *CTLA-4* -1722T/C polymorphism among the controls were tested for Hardy–Weinberg equilibrium (HWE) using an internet-based calculator (http://ihg.gsf.de/cgi-bin/hw/hwa1.pl). The associations between *CTLA-4* -1722T/C locus and the risk of ESCC were analyzed by unconditional logistic regression for crude ORs and adjusted ORs when it was appropriate. Statistical analyses were implemented in SAS 9.1.3 software (SAS Institute, Cary, NC). A *P*<0.05 (two-tailed) was defined as the criterion of statistical significance.

### Meta analysis

The meta-analysis is reported on the basis of the Preferred Reporting Items for Meta-analyses (PRISMA) guideline (**Checklist S1**) [13].

Embase, PubMed, and CBM (Chinese BioMedical Disc), as well as CNKI (China National Knowledge Infrastructure) database were searched up to August 1st, 2013 for publications investigating the association of *CTLA-4* -1722T/C polymorphism with cancer risk. The combination terms were ‘cancer’ or ‘tumor’ or ‘carcinoma’ or ‘neoplasm’ and ‘cytotoxic T-lymphocyte antigen 4′ or ‘*CTLA-4*′ or ‘CD152’, annexed with ‘mutation’ or ‘variant’ or ‘SNP’ or ‘polymorphism’. In addition, the publication language was restricted to English and Chinese, and all studies performed in human subjects were identified. The search results were supplemented by checking all references listed in these studies and published reviews. Included studies were qualified if they met the major included criteria: (1) designed as a retrospective or nested case-control study, (2) evaluated the *CTLA-4* -1722T/C polymorphism and cancer risk, (3) provide genotype counts of *CTLA-4* -1722T/C polymorphism between cancer cases and controls, and (4) control genotype distributions consistent with HWE. The major excluded criteria were: (1) not case-control studies, (2) review publications and (3) overlapping data. Information was carefully and independently extracted by three reviewers (W. Tang, H. Qiu, and H. Jiang). In case of conflicting evaluations, differences were resolved by further discussion among all authors. The following data was extracted: first author, year of publication, cancer type, country, ethnicity, number of cases and controls, genotype method, allele and genotype frequency, and HWE in controls.

In this meta-analysis, the crude odds ratio (OR) with the corresponding 95% confidence intervals (95% CI) was used to assess the strength of association between the *CTLA-4* -1722T/C polymorphism and cancer risk. The Z-test and *P*-value (two-tailed) was used to measure the significance of the pooled OR, and statistical significance was defined as *P*<0.05 (two-tailed). Heterogeneity among studies was evaluated by a Chi-square-based I^2^ test, I^2^<25% indicated low heterogeneity, 25%≤I^2^≤50% indicated moderate heterogeneity, and I^2^>50% indicated large heterogeneity [14]. If I^2^>50% or *P*<0.10, the pooled ORs were calculated by the random-effects model (the DerSimonian–Laird method), otherwise the fixed-effects model was implemented (the Mantel–Haenszel method). Subgroup analyses were implemented to measure ethnicity-specific, cancer type-specific and system-specific effects according to ethnicity, cancer type (if any cancer type evaluated by less than three individual investigations, it was combined into "other cancers") and system. The funnel plot and Egger's test were carried out to measure publication bias, which was evaluated by visual inspection of an asymmetric plot. For heterogeneity, funnel plot and Egger's test, statistical significance was considered at *P*<0.1. In this meta-analysis, all statistical analyses were conducted by STATA software (version 12.0).

## Results

### Baseline characteristics

The demographics and risk factors of all subjects are presented in **Table 1**. The results indicated that cases and controls were fully matched by age and gender. However, there was significant difference on drinking status and smoking between patients and controls (*P*<0.001). The primary information of *CTLA-4* -1722T/C polymorphism was showed in **Table 2**. For this SNP, the genotyping success rate was 96.43% in all samples. Minor allele frequency (MAF) of controls in our study, was similar to the database of Chinese for this SNP (**Table 2**). The genotypic frequencies for *CTLA-4* -1722T/C polymorphism among controls were used to evaluated deviation from the HWE, and the result was in HWE (*P* = 0.284) (**Table 2**).

**Table 2:** Primary information for *CTLA4* -1722T/C (rs733618) polymorphism.

Genotyped SNPs	*CTLA4* -1722T/C (rs733618)
Chromosome	2
Function	nearGene-5
Chr Pos (Genome Build 36.3)	204439189
Regulome DB Score^a^	No Data
TFBS^b^	Y
Splicing (ESE or ESS)	—
miRNA (miRanda)	—
nsSNP	—
MAF^c^ for Chinese in database	0.390
MAF in our controls (n = 686)	0.414
*P* value for HWE^d^ test in our controls	0.701
Genotyping method^e^	LDR
% Genotyping value	96.43%

a http://www.regulomedb.org/;

b TFBS: Transcription Factor Binding Site (http://snpinfo.niehs.nih.gov/snpinfo/snpfunc.htm);

c MAF: minor allele frequency;

d HWE: Hardy–Weinberg equilibrium;

e LDR: Ligation Detection Reaction.

### Single-locus analysis

In the single locus analyses, the genotype frequencies of *CTLA-4* -1722T/C were 16.53% (CC), 49.10% (TC) and 34.37% (TT) in the patients, and 17.50% (CC), 47.79% (TC) and 34.70% (TT) in the controls, and the difference was no statistically significant (*P* = 0.862) (**Table 3**). In this case-control study, logistic regression analyses showed that the *CTLA-4* -1722T/C SNP was not associated with the risk of ESCC. Tobacco use and alcohol consumption are two strong environmental factors, we examined the association in a stratified analysis by these two factors and the results were null association (**Table 4**).

**Table 3:** Logistic regression analyses of associations between *CTLA4* -1722T/C (rs733618) polymorphisms and risk of ESCC.

Genotype	Cases (n = 629)		Controls (n = 686)		Crude OR (95%CI)	*P*	Adjusted OR a (95%CI)	*P*
	n	%	n	%				
*CTLA4* rs733618T/C								
TT	210	34.37	228	34.70	1.00		1.00	
TC	300	49.10	314	47.79	1.04 (0.81–1.33)	0.770	1.06 (0.83–1.37)	0.625
CC	101	16.53	115	17.50	0.95 (0.69–1.32)	0.776	0.97 (0.69–1.35)	0.846
CC vs. TC vs. TT						0.862		
TC+CC	401	65.63	429	65.30	1.02 (0.81–1.28)	0.901	1.04 (0.82–1.32)	0.755
TT+TC	510	83.47	542	82.50	1.00		1.00	
CC	101	16.53	115	17.50	0.93 (0.70–1.25)	0.645	0.93 (0.69–1.26)	0.649
T allele	720	58.92	770	58.60	0.99 (0.84–1.16)	0.870		
C allele	502	41.08	544	41.40				

a Adjusted for age, sex, smoking and drinking status; Bold values are statistically significant (*P*<0.05).

**Table 4:** Stratified analyses between *CTLA4* -1722T/C (rs733618) polymorphism and ESCC risk by sex, age, smoking status and alcohol consumption.

Variable	*CTLA4* rs733618 T/C (case/control)^a^				Adjusted OR^b^ (95% CI); *P*				
	TT	TC	CC	TC+CC	TT	TC	CC	TC+CC	CC vs. (TC+TT)
Sex									
Male	150/154	209/214	70/76	279/290	1.00	1.04 (0.77–1.40); *P*: 0.815	0.96 (0.64–1.43); *P*: 0.828	1.02 (0.76–1.35); *P*: 0.916	0.94 (0.65–1.35); *P*: 0.723
Female	60/74	91/100	31/39	122/139	1.00	1.10 (0.70–1.72); *P*: 0.676	1.02 (0.57–1.83); *P*: 0.955	1.08 (0.71–1.64); *P*: 0.731	0.96 (0.57–1.63); *P*: 0.888
Age									
<63	102/125	139/162	60/60	199/222	1.00	1.05 (0.74–1.51); *P*: 0.773	1.24 (0.79–1.96); *P*: 0.353	1.11 (0.79–1.55); *P*: 0.559	1.21 (0.80–1.82); *P*: 0.371
≥63	108/103	161/152	41/55	202/207	1.00	1.05 (0.73–1.49); *P*: 0.807	0.73 (0.45–1.20); *P*: 0.214	0.96 (0.69–1.35); *P*: 0.820	0.71 (0.46–1.11); *P*: 0.136
Smoking status									
Never	108/171	185/218	54/85	239/303	1.00	1.31 (0.96–1.80); *P*: 0.092	0.99 (0.65–1.52); *P*: 0.963	1.22 (0.91–1.65); *P*: 0.190	0.84 (0.58–1.24); *P*: 0.380
Ever	102/57	115/96	47/30	162/126	1.00	0.71 (0.46–1.10); *P*: 0.123	0.91 (0.51–1.62); *P*: 0.749	0.76 (0.50–1.14); *P*: 0.187	1.11 (0.66–1.86); *P*: 0.693
Alcohol consumption									
Never	145/178	208/231	63/91	271/322	1.00	1.17 (0.87–1.58); *P*: 0.300	0.89 (0.59–1.33); *P*: 0.563	1.09 (0.82–1.45); *P*: 0.548	0.81 (0.56–1.17); *P*: 0.257
Ever	65/50	92/83	38/24	130/107	1.00	0.81 (0.50–1.32); *P*: 0.399	1.20 (0.63–2.29); *P*: 0.577	0.90 (0.57–1.42); *P*: 0.648	1.36 (0.76–2.43); *P*: 0.296

a The genotyping was successful in 611 (97.1%) ESCC cases, and 657 (95.8%) controls for *CTLA4* -1722T/C (rs733618);

b Adjusted for age, sex, smoking status and alcohol consumption (besides stratified factors accordingly) in a logistic regression model.

### Eligible articles for meta-analysis

**Figure 1:**
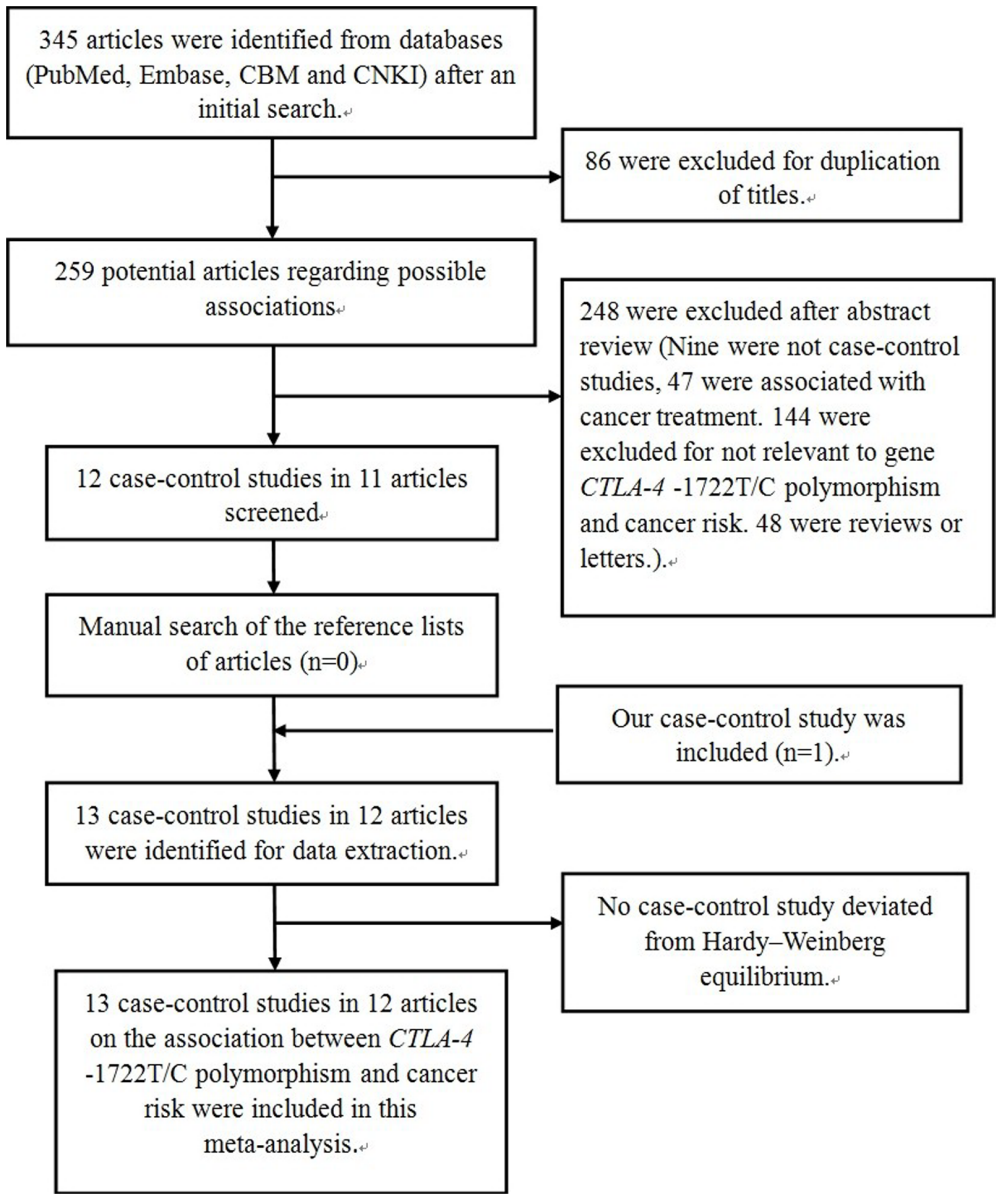
Flow diagram of articles selection process for *CTLA-4* -1722T/C (rs733618) polymorphism and cancer risk meta-analysis.

The initial search yielded a total of 345 potentially relevant publications. After applying additional filters, 12 case-control studies in 11 publications and our study were eligible for inclusion. The detailed process of selecting and excluding articles is presented in **Figure 1**.

### Study characteristics

There were two groups in an article conducted by Hadinia et al. [15], we treated them separately. In total 12 separate studies plus our case-control study involving a total of 3420 cancer cases and 3675 controls were included in this meta-analysis. Among the 13 case-control studies, three investigated breast cancer [16]–[18], three investigated gastric cancer [15], [19], [20], and the other studies investigated cervical cancer, lung cancer, esophageal cancer, colorectal cancer, and oral cancer [6], [15], [21]–[24]. As for subjects in these studies, 8 were Asians [6], [17]–[21], [24] and 5 were Caucasians[15], [16] [22], [23]. Characteristics of each included study are presented in **Table 5**. The detailed distribution of the *CTLA-4* -1722T/C polymorphism and allele among cases and controls is presented in **Table 6**.

**Table 5:** Characteristics of populations and cancer types of the individual studies included in the meta-analysis.

study	year	country	ethnicity	cancer type	No. of cases/controls	Genotype Method
Bharti et al.	2013	India	Asians	oral cancer	130/180	PCR-RFLP
Li et al.	2012	China	Asians	breast cancer	581/566	PCR-RFLP
Qi et al.	2012	China	Asians	gastric cancer	118/96	PCR-RFLP
Jiang et al.	2011	China	Asians	cervical cancer	100/100	MALDI-TOF-MS
Khaghanzadeh et al.	2010	Iran	Caucasians	lung cancer	127/124	PCR-RFLP, PCR-ARMS
Rahimifar et al.	2010	Iran	Caucasians	cervical cancer	55/110	PCR-RFLP, PCR-ARMS
Li et al.	2008	China	Asians	breast cancer	328/327	PCR-RFLP
Sun et al.	2008	China	Asians	lung cancer	765/800	PCR-RFLP, MALDI-TOF MS
Hadinia et al.	2007	Iran	Caucasians	gastric cancer	46/190	RFLP, PCR-ARMS
Hadinia et al.	2007	Iran	Caucasians	colorectal cancer	109/190	RFLP, PCR-ARMS
Song et al.	2006	China	Asians	gastric cancer	183/116	PCR-RFLP
Erfani et al.	2006	Iran	Caucasians	breast cancer	283/245	PCR-CTPP
Our study	2013	China	Asians	esophageal cancer	629/686	PCR-LDR

MALDI–TOF–MS: Matrix-Assisted Laser Desorption/Ionization Time of Flight Mass Spectrometry.

PCR-RFLP: polymerase chain reaction-restriction fragment length polymorphism.

PCR-LDR: polymerase chain reaction-ligase detection reaction.

PCR-ARMS: AmplificationRefractory Mutation System-Polymerase Chain Reaction.

**Table 6:** Distribution of *CTLA-4* -1722T/C (rs733618 T/C) polymorphisms genotype and allele among multiple cancer patients and controls.

		case			control			case		control		HWE,*P* value
study	year	TT	TC	CC	TT	TC	CC	c	T	C	T	
Qi et al.	2012	40	69	9	37	45	14	87	149	73	119	0.957723
Li et al.	2012	184	276	114	207	256	88	504	644	432	670	0.552314
Jiang et al.	2011	37	49	14	43	39	18	77	123	75	125	0.092957
Rahimifar et al.	2010	46	8	1	90	20	0	10	100	20	200	0.294266
Khaghanzadeh et al.	2010	106	19	1	98	16	1	21	231	18	212	0.702320
Sun et al.	2008	719	43	3	762	37	1	49	1481	39	1561	0.435355
Li et al.	2008	125	163	40	111	168	48	243	413	264	390	0.224758
Hadinia et al.(colorectal)	2007	97	12	0	165	24	0	12	206	24	354	0.351131
Hadinia et al.(gastric)	2007	42	4	0	165	24	0	4	88	24	354	0.351131
Erfani et al.	2006	225	54	3	204	41	0	60	504	41	449	0.152921
Bharti et al.	2013	92	25	6	131	46	3	37	209	52	308	0.648604
Song et al.	2006	62	113	8	45	54	17	129	237	88	144	0.902590
Our study	2013	210	300	101	228	314	115	502	720	544	770	0.700586

HWE: Hardy–Weinberg equilibrium.

### Meta-analysis results

**Figure 2:**
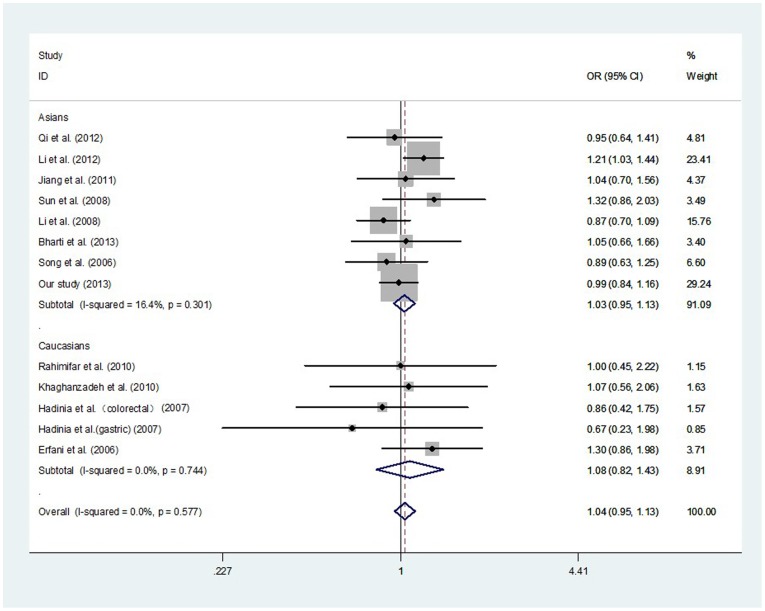
Meta-analysis with a fixed-effects model for the association between the risk of cancer and the *CTLA-4* -1722T/C polymorphism (C vs. T).

**Figure 3:**
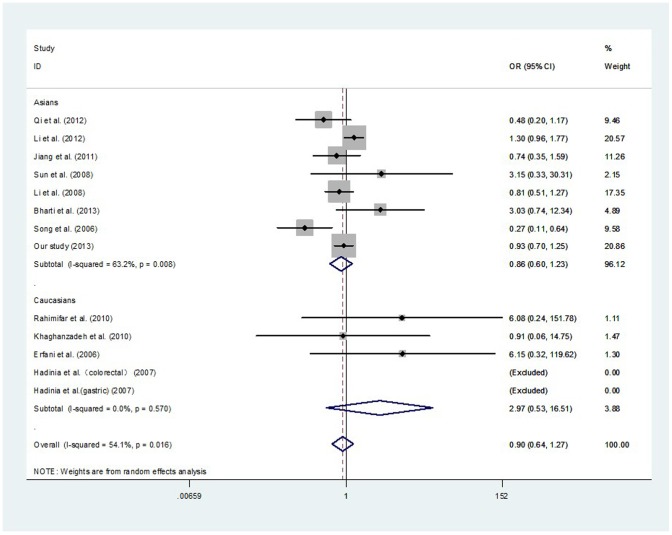
Meta-analysis with a random-effects model for the association between the risk of cancer and the *CTLA-4* -1722T/C polymorphism (CC vs. TC+TT).

After combining all qualified studies, a total of 3420 cancer cases and 3675 controls from 13 eligible case–control studies were included for meta-analysis of the association between the CTLA-4 -1722T/C polymorphism and cancer risk. There was null association of *CTLA-4* -1722T/C polymorphism with overall cancer risk in all genetic models (**Table 7** **,** **Table 8** **,** **Table 9** **,** **Figure 2** **, and** **Figure 3**). In a stratified analysis by ethnicity, the similar results were observed in both Asians and Caucasians (**Table 7**). In a stratified analysis by cancer type, there was a decreased risk of gastric cancer in two genetic models: CC vs. TC+TT (OR, 0.36; 95% CI, 0.19–0.66; *P* = 0.001) and CC vs. TT (OR, 0.45; 95% CI, 0.23–0.86; *P* = 0.016) (**Table 8**). In a stratified analysis by system, null association was also observed (**Table 9**).

**Table 7:** Summary of results of the meta-analysis from different comparative genetic models in the subgroup analysis by ethnicity.

Polymorphism	Genetic comparison	Population	OR(95%CI); *P*	Test of heterogeneity	
				(*p* -Value, I^2^)	Model
	CC+TC vs. TT	All	1.09(0.97–1.22);0.159	0.762,0.0%	F
		Asians	1.09(0.97–1.24);0.160	0.494,0.0%	F
		Caucasians	1.04(0.78–1.41);0.773	0.767,0.0%	F
	CC vs. TC+TT	All	0.90(0.64–1.27);0.553	0.016,54.1%	R
		Asians	0.86(0.60–1.23);0.400	0.008,63.2%	R
		Caucasians	3.27(0.65–16.32);0.149	0.570,0.0%	F
*CTLA-4* -1722T/C	CC vs. TT	All	0.98(0.70–1.37);0.906	0.050,45.3%	R
		Asians	0.94(0.66–1.33);0.719	0.028,55.4%	R
		Caucasians	3.29(0.66–16.46);0.146	0.575,0.0%	F
	TC vs. TT	All	1.09(0.97–1.23);0.154	0.641,0.0%	F
		Asians	1.11(0.97–1.26);0.124	0.358,9.3%	F
		Caucasians	1.01(0.74–1.36);0.970	0.792,0.0%	F
	C vs. T	All	1.04(0.95–1.13);0.383	0.577,0.0%	F
		Asians	1.03(0.95–1.13);0.460	0.301,16.4%	F
		Caucasians	1.08(0.82–1.43);0.575	0.744,0.0%	F

F indicates fixed model; R indicates random model.

**Table 8:** Summary of results of the meta-analysis from different comparative genetic models in the subgroup analysis by cancer type.

Polymorphism	Genetic comparison	Cancer type	OR(95%CI); *P*	Test of heterogeneity	
				(*p* -Value, I^2^)	Model
	CC+TC vs. TT	All	1.09(0.97–1.22);0.159	0.762,0.0%	F
		Gastric cancer	1.15(0.81–1.62);0.430	0.571,0.0%	F
		Breast cancer	1.10(0.83–1.47);0.514	0.100,56.5%	R
		Other cancers	1.05(0.89–1.24);0.589	0.903,0.0%	F
	CC vs. TC+TT	All	0.90(0.64–1.27);0.553	0.016,54.1%	R
		Gastric cancer	**0.36(0.19–0.66);0.001**	0.347,0.0%	F
		Breast cancer	1.10(0.68–1.77);0.689	0.121,52.7%	R
		Other cancers	0.98(0.76–1.28);0.903	0.374,6.6%	F
*CTLA-4*-1722T/C	CC vs. TT	All	0.98(0.70–1.37);0.906	0.050,45.3%	R
		Gastric cancer	**0.45(0.23–0.86);0.016**	0.412,0.0%	F
		Breast cancer	1.15(0.60–2.22);0.672	0.046,67.6%	R
		Other cancers	1.04(0.78–1.39);0.798	0.496,0.0%	F
	TC vs. TT	All	1.09(0.97–1.23);0.154	0.641,0.0%	F
		Gastric cancer	1.34(0.94–1.91);0.107	0.392,0.0%	F
		Breast cancer	1.09(0.90–1.31);0.383	0.259,25.9%	F
		Other cancers	1.04(0.88–1.24);0.637	0.741,0.0%	F
	C vs. T	All	1.04(0.95–1.13);0.383	0.577,0.0%	F
		Gastric cancer	0.90(0.70–1.15);0.406	0.833,0.0%	F
		Breast cancer	1.09(0.85–1.41);0.504	0.044,68.0%	R
		Other cancers	1.02(0.90–1.16);0.733	0.931,0.0%	F

F indicates fixed model; R indicates random model.

**Table 9:** Summary of results of the meta-analysis from different comparative genetic models in the subgroup analysis by system.

Polymorphism	Genetic comparison	Cancer type	OR(95%CI); *P*	Test of heterogeneity	
				(*p* -Value, I^2^)	Model
	CC+TC vs. TT	All	1.09(0.97–1.22);0.159	0.762,0.0%	F
		Digestive system cancer	1.02(0.86–1.22);0.797	0.839,0.0%	F
		Reproductive and breast cancer	1.12(0.95–1.32);0.186	0.275,22.0%	F
		Respiratory system cancer	1.22(0.84–1.78);0.288	0.697,0.0%	F
	CC vs. TC+TT	All	0.90(0.64–1.27);0.553	0.016,54.1%	R
		Digestive system cancer	0.71(0.33–1.53);0.381	0.008,74.5%	R
		Reproductive and breast cancer	1.11(0.88–1.40);0.395	0.171,37.5%	F
		Respiratory system cancer	1.99(0.37–10.85);0.425	0.498,0.0%	F
*CTLA-4*-1722T/C	CC vs. TT	All	0.98(0.70–1.37);0.906	0.050,45.3%	R
		Digestive system cancer	0.79(0.41–1.52);0.476	0.056,60.3%	R
		Reproductive and breast cancer	1.18(0.91–1.53);0.217	0.111,46.7%	F
		Respiratory system cancer	2.02(0.37–10.99);0.417	0.499,0.0%	F
	TC vs. TT	All	1.09(0.97–1.23);0.154	0.641,0.0%	F
		Digestive system cancer	1.06(0.88–1.27);0.529	0.386,4.8%	F
		Reproductive and breast cancer	1.10(0.92–1.31);0.289	0.392,2.6%	F
		Respiratory system cancer	1.19(0.81–1.75);0.367	0.791,0.0%	F
	C vs. T	All	1.04(0.95–1.13);0.383	0.577,0.0%	F
		Digestive system cancer	0.96(0.85–1.09);0.569	0.966,0.0%	F
		Reproductive and breast cancer	1.09(0.96–1.23);0.168	0.175,37.0%	F
		Respiratory system cancer	1.24(0.87–1.78);0.232	0.595,0.0%	F

F indicates fixed model; R indicates random model.

### Tests for publication bias, sensitivity analyses, and heterogeneity

**Figure 4:**
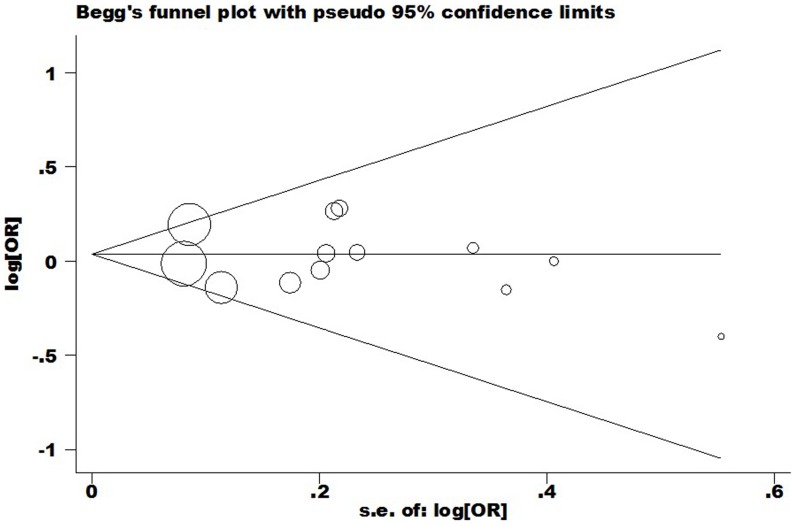
Begg's funnel plot of meta-analysis of between the *CTLA-4* -1722T/C polymorphism and the risk of cancer (fixed–effects estimates) (C vs. T compare genetic model).

In this meta-analysis, potential publication bias was detected by Begg's Funnel plot and Egger's test (**Figure 4**), and the shape of funnel was symmetry in all genetic model. It suggested that there were no publication bias for overall cancer in this meta-analysis (C vs. T: Begg's test *P* = 0.855, Egger's test *P* = 0.675; CC vs. TT: Begg's test *P* = 0.350, Egger's test *P* = 0.709; TC vs. TT: Begg's test *P* = 0.583, Egger's test *P* = 0.702; CC+TC vs. TT: Begg's test *P* = 0.161, Egger's test *P* = 0.576; CC vs. TT+TC: Begg's test *P* = 0.533, Egger's test *P* = 0.845).

**Figure 5:**
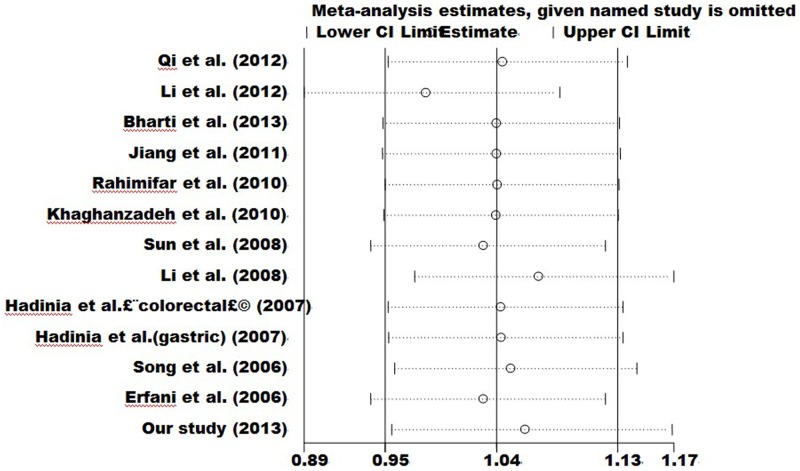
Sensitivity analysis of the influence of C vs. T in overall cancer meta–analysis (fixed–effects estimates).

Sensitivity analyses were carried out to detect the influence of each individual dataset on the pooled OR, with each study dataset set dropped at a time. The outcomes did not change when any individual study was omitted, suggesting the stability of our results (**Figure 5**) (data not shown).

**Figure 6:**
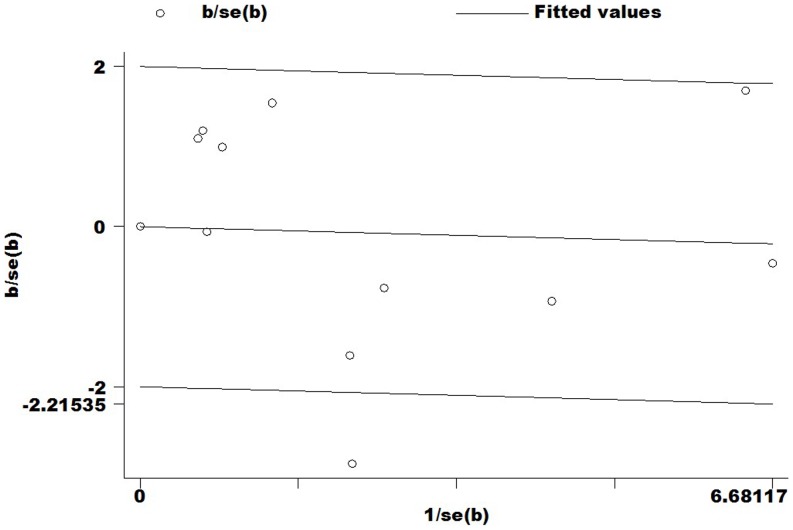
Galbraith radial plot of meta–analysis (CC vs. TC+TT compare genetic model).

Large heterogeneities among the studies were indentified in the recessive model and homozygous model. Since tumor origin, ethnicity and system can influence the results from meta–analyses, we carried out subgroup analyses and the results were presented in **Table 7** **,** **Table 8** and **Table 9**. The results indicated that breast cancer, digestive system cancer and Asian population subgroup may contribute to the major heterogeneity. As shown in **Table 7**, heterogeneity was significant in the recessive model. Further analysis was conducted by Galbraith radial plot in the recessive model (**Figure 6**), and the result showed one outlier might contribute to the major sources of heterogeneity. From the forest plot in the recessive model (**Figure 2**), one can identify that a case-control study conducted by Erfani et al.[16] contributes the main heterogeneity.

## Discussion

Of late, several studies have investigated the association between *CTLA-4* -1722T/C polymorphism and multiple cancers, a decisive answer is lacking. In this study, a case-control study in Han Chinese population, along with a meta-analysis on overall cancer, attempted to derive a comprehensive evaluation and the results were non-significance. To the best of our knowledge, this is the first case-control study investigating the association between *CTLA-4* -1722T/C polymorphism and esophageal cancer risk.

Cancer and autoimmune disease are both multifactorial disorders that results from complex interactions between genetic backgrounds and environmental factors. The *CTLA-4* -1722T/C polymorphism (T→C) would reduce a transcription factor binding site for nuclear factor 1 and weaken the expression of cell surface CTLA-4 [11], [25], which might play an important role in cancer and autoimmune disease susceptibility. Several meta-analyses showed that *CTLA-4* -1722T/C polymorphism might be a risk factor for systemic lupus erythematosus susceptibility [26]–[29]. However, the association between this locus and cancer risk was inconclusive. With a growing interest in the associations of genetic polymorphisms and cancer, several studies have examined the hypothesis that *CTLA-4* -1722T/C polymorphism is relevant to the risk of a number of cancers; however, the results remain elusive. Considering the fact that most common SNPs usually make low penetrance cancer susceptibility, this study includes 13 case-control studies with relatively large sample sizes to obtain a precise evaluation between *CTLA-4* -1722T/C genetic variation and cancer risk. One individual study has reported positive signal of *CTLA-4* -1722T/C polymorphism with cancer [18]; the other individual study has reported negative signal [20]; however, as demonstrated in our overall genetic model results among 7098 subjects, there were non-significance, even in different population subgroups and different system. In a stratified analysis by cancer type, the protective effect conferred by the recessive model and homozygous model was appreciably obvious in gastric cancer subgroup. Considering only three case-control studies were conducted in gastric cancer subgroup and these studies were small sample sizes, which might restrict power to confirm a real influence or generate a fluctuated assessment. All results should be interpreted with very caution. It is also possible that the potential function of this polymorphism is diluted or covered by other genetic background or environment factors, and these important factors should not be ignored. Considering only 13 case-control studies were recruited in this meta-analysis and most of these studies were small sample sizes, in the future, further investigations with large sample sizes should be carried out to confirm or refute these results.

Some merit of current study should be adequate consideration. First, this is to date the first case-control study detecting the association of *CTLA-4* gene -1722T/C polymorphism with esophageal cancer. Second, the findings of our case-control study conform to that of the subsequent meta-analysis. Third, in our case-control study, control genotype distributions were consistent with HWE showed our results were less prone to selection bias, the shape of funnel plot indicated that there were no publication bias in current meta-analysis. Fourth, relatively low heterogeneity was observed between publications for *CTLA-4* -1722T/C polymorphism.

In addition, some limitations in current study should be acknowledged when interpreting our results. First, in this case-control study, all cases and controls were recruited from two hospitals and might not fully represent the general Chinese populations. Second, all included case–control studies for meta-analysis were from Asians and Caucasians; thus, our findings might only be suitable for these two populations. Third, only published studies were recruited in this meta-analysis, publication bias might have inevitably occurred. Fourth, due to the lack of uniform background data for recruited studies, data were not further stratified by other factors (such as, age, gender, smoking, alcohol consumption, and other lifestyle factors). Fifth, in this study, we focused on only -1722T/C polymorphism in *CTLA-4*, and did not consider other susceptibility genes or polymorphisms. For the low penetrance cancer susceptibility gene effects from SNP, these important genetic and environmental factors should be adequately considered.

In summary, this case-control study along with a meta-analysis, failed to confirm the association between *CTLA-4* -1722T/C polymorphism and cancer risk, even across different ethnic subgroups and different systems. In the future, further investigations with large sample sizes and detailed gene–environment data, should be carried out to confirm or refute these results.

## Supporting Information

Figure S1 Direct sequencing analyses for genotypes of CTLA-4 -1722T/C SNP (The three charts represent three genotypes).
(TIF)

Checklist S1 PRISMA checklist, Checklist of items to include when reporting a systematic review or meta-analysis (diagnostic review consisting of cohort studies).
(DOCX)
