# Supplementary figures and images for: Lack of Association between Cytotoxic T-lymphocyte Antigen 4 (CTLA-4) -1722T/C (rs733618) Polymorphism and Cancer Risk: From a Case-Control Study to a Meta-Analysis
Source: PLoS One. 2014 Apr 7;9(4):e94039. doi: 10.1371/journal.pone.0094039 (PMC3978075; doi:10.1371/journal.pone.0094039)

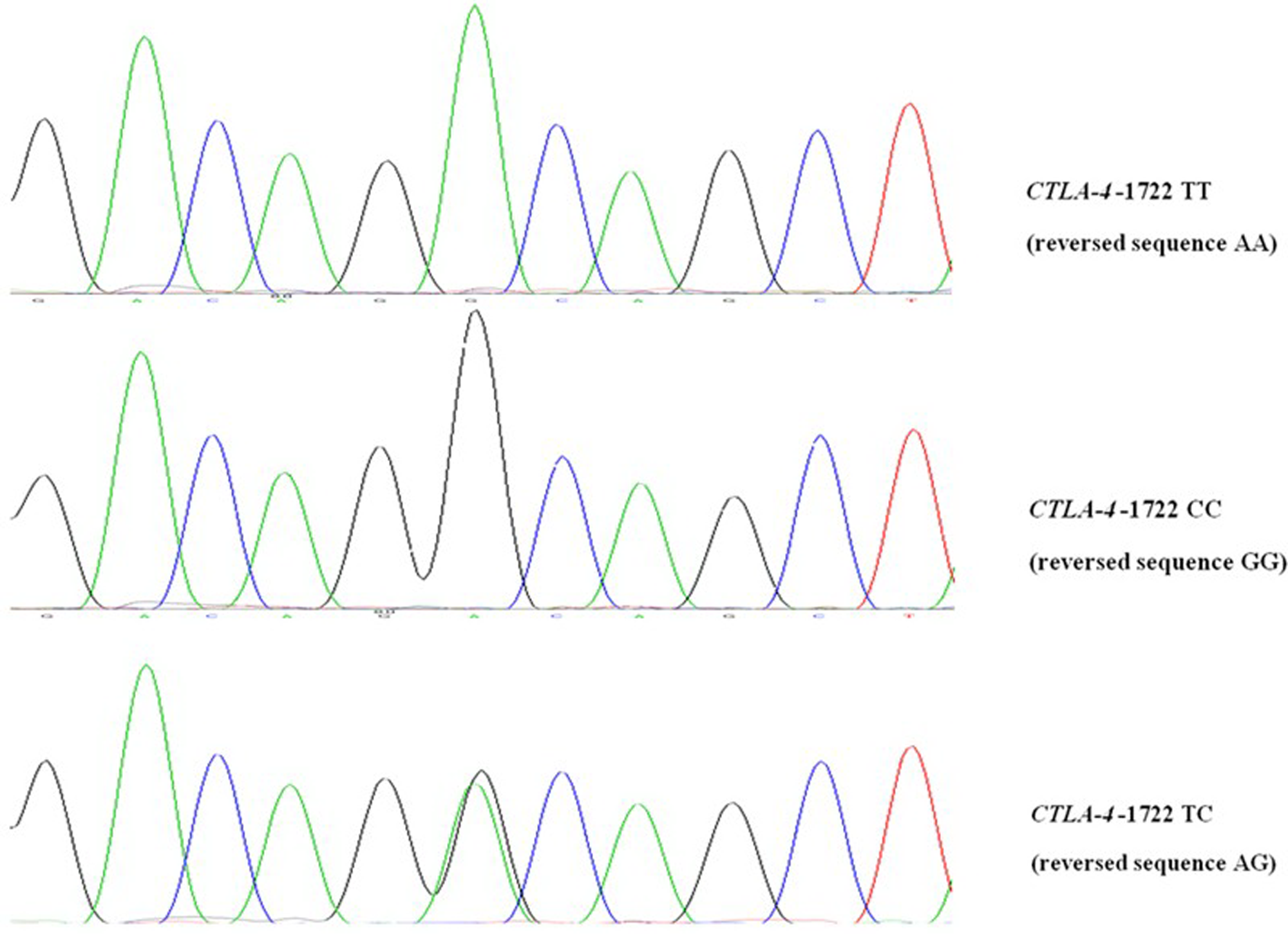

Supplement: Figure S1 — Direct sequencing analyses for genotypes of CTLA-4 -1722T/C SNP (The three charts represent three genotypes). (TIF) [file pone.0094039.s001.tif]
